# Perceptual changes after learning of an arbitrary mapping between vision and hand movements

**DOI:** 10.1038/s41598-022-15579-8

**Published:** 2022-07-06

**Authors:** Wladimir Kirsch, Wilfried Kunde

**Affiliations:** Department of Psychology, University of Würzburg, Röntgenring 11, 97070 Würzburg, Germany

**Keywords:** Psychology, Human behaviour, Somatosensory system, Visual system, Perception, Attention

## Abstract

The present study examined the perceptual consequences of learning arbitrary mappings between visual stimuli and hand movements. Participants moved a small cursor with their unseen hand twice to a large visual target object and then judged either the relative distance of the hand movements (Exp.1), or the relative number of dots that appeared in the two consecutive target objects (Exp.2) using a two-alternative forced choice method. During a learning phase, the numbers of dots that appeared in the target object were correlated with the hand movement distance. In Exp.1, we observed that after the participants were trained to expect many dots with larger hand movements, they judged movements made to targets with many dots as being longer than the same movements made to targets with few dots. In Exp.2, another group of participants who received the same training judged the same number of dots as smaller when larger rather than smaller hand movements were executed. When many dots were paired with smaller hand movements during the learning phase of both experiments, no significant changes in the perception of movements and of visual stimuli were observed. These results suggest that changes in the perception of body states and of external objects can arise when certain body characteristics co-occur with certain characteristics of the environment. They also indicate that the (dis)integration of multimodal perceptual signals depends not only on the physical or statistical relation between these signals, but on which signal is currently attended.

## Introduction

How observers perceive their own body is influenced not only by sensations from inside their body (i.e. by interoception^1^) but also by information from the environment (i.e. exteroception). For example, when wearing prism glasses, the felt position of the hand shifts towards where the hand looks to be^2^. Conversely, how we perceive our environment is influenced by interoceptive states of the observer. The perceived orientation of a visual stimulus, e.g., is biased towards the current body orientation when that stimulus is judged relative to gravity and the body is tilted^3^.

Such intersensory biases likely result from integration of multimodal signals which is constrained by several experimental factors^4^. Most importantly, the magnitude of integration is determined by the observer’s believe that signals belong to the same object or event, the so called “unity assumption”: The more evidence suggests that signals from different senses relate to the same event, the stronger is their integration^5–10^. For example, consider an observer who is confronted with auditory and visual signals, such as short sound bursts and light flashes, presented at different spatial locations and at different points in time. When asked to report whether the visual and auditory stimuli originate from the same or different spatial locations, observer’s judgments indicating the same spatial origin decrease when spatial or temporal discrepancy between visual and auditory stimuli increase. Simultaneously, the impact of one modality on another, such as a bias of auditory perception (of location) by visual signals, that indicates audio-visual integration decreases when the spatial or temporal conflict between vision and audition gets larger^11^, see also Ref.^9^ for related observations.

Sensory integration accounts predict perceptual changes if there is a discrepancy between multimodal signals. For example, if an object is simultaneously seen and manually grabbed the perceived size of the object is usually in-between the visual and haptic sizes^12,13^. Thus, the visual perception of the object is biased towards the haptic signal and vice versa, the haptic perception is biased towards the visual signal. However, intersensory discrepancies sometimes entail biases of the opposite direction. For example, when asked to localize an auditory stimulus, participants’ reports of object location are biased away from visual stimuli presented in the vicinity of the auditory stimulus when they believe that the origins of both types of stimuli do not coincide^11^. Such a pattern indicates that the signals are not integrated.

Several studies showed that multimodal signals are combined in perception although they are related to spatially clearly separated objects and events^14–18^. In a virtual grasping task, e.g., we presented a visual object in the fronto-parallel plane of the observer and asked her/him to enclose this object by a pair of manually controlled visual cursors^17^. We then measured the perceived visual size of the object and the felt finger posture (i.e. hand opening). A typical result in this paradigm is that the perceived hand opening is biased by the size of the object and vice versa, the perceived size of the object is biases by the actual hand opening (though to lesser extent) when there is a spatial discrepancy between hand opening and object size. These observations suggest that a strict “object unity” is not a mandatory condition for multisensory integration to occur, but instead, that some sort of correlation or spatio-temporal correspondence suffices.

Altogether there is evidence for various possible changes in the perception of observers’ bodies and their environment with intersensory discrepancy. This discrepancy between modalities can only be induced when a certain correspondence or mapping between them already exists. In the present study, we wondered about whether it is possible to learn two arbitrary mappings between vision and hand movements during a virtual interaction with a distant object and whether this can cause changes in the perception of the hand movements and of the external object when an intersensory discrepancy is introduced. Previous studies already demonstrated that sensory signals from different modalities can be arbitrarily paired^19–22^. The present study expands the scope of this research by situations where multimodal signals have clearly different origins (i.e. relate to spatially separated objects).

This rather specific multisensory issue can also be informative for other related research directions. For example, several observations indicated an impact of the perceiver’s body and its action on the perception of external objects. Hills were judged as steeper when wearing a heavy backpack, golf players who played well judged holes as larger than players who played less well, parkour experts judged walls as smaller than parkour novices, to name a few examples see Ref.^23,24^ for reviews, and Ref.^25^ for criticism. We have suggested that such phenomena could be outcomes of multisensory integration between vision and body-related (e.g. proprioceptive) signals by analogy with any other modality combination^26,27^. The present study can be considered as an additional test of this claim. To put it differently, the basic question of the present study was whether it is possible to change perception (e.g. of hill steepness, hole size, or wall height), by observers experiencing a systematic relation between their body movement and an external object (by repeatedly ascending hills, shooting golf balls, or jumping over walls) while a change (or a kind of discrepancy) in this intersensory relation is introduced (i.e. by comparing hill slop judgements with or without a backpack, successful with unsuccessful gulf puts, experts with novices etc.). While in this sort of research perception is biased based on some pre-experimentally established linkages between motor output and perceptual feedback we here want to study if such perceptual biases can be instantiated by novel and deliberately arbitrary linkages encountered in the experiment alone.

**Figure 1:**
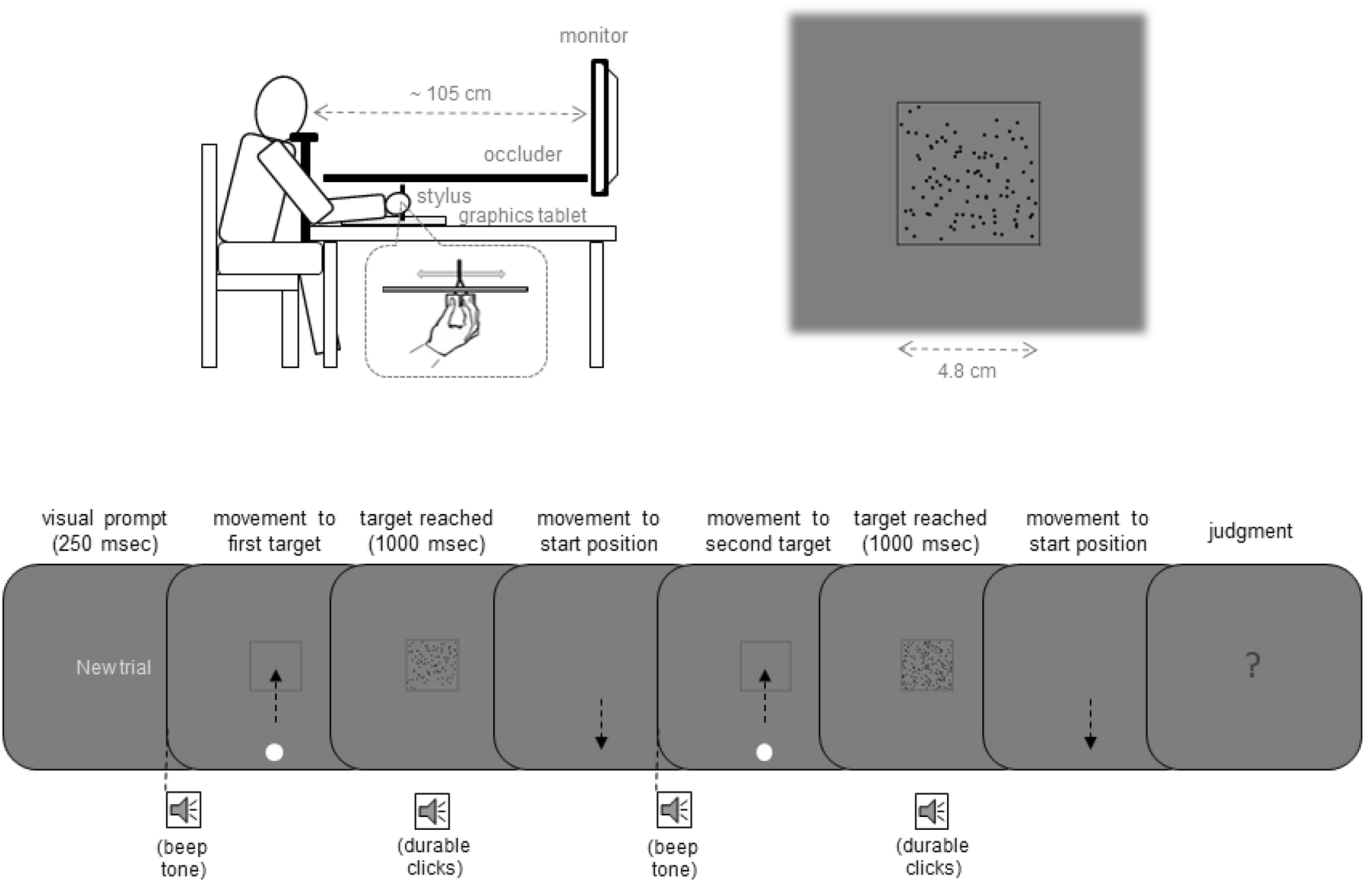
Experimental setup (upper part) and main trial events (lower part) in Experiments 1 and 2. Participants performed a linear hand movement on a horizontal plane with their right hand that controlled the movement of a visual cursor on a vertical plane following a go signal (“beep tone”). When the center of a square was reached by the cursor, the square was filled with a number of dots while an acoustic signal was presented (“durable clicks” that served as additional feedback that the movement goal was reached). After a backward movement, another goal directed movement was performed. Following the second backward movement, participants indicated whether the first or the second hand movement covered a larger distance (Experiment 1) or whether the first or the second square contained more dots (Experiment 2) by pressing a mouse button.

In the present study, participants moved a cursor to a square shown in the center of a screen by moving a stylus on a graphics tablet (see Fig. 1). Then the square was filled with a number of randomly distributed dots. This occurred two times within a single trial and the participants were asked to estimate either which hand movement covered a larger distance (Experiment 1) or which square had more dots (Experiment 2). We varied the mapping between hand movement distance and the number of dots so that an increase in movement distance was associated either with an increase (Group A) or with a decrease (Group B) in the number of dots. Following a learning phase, we introduced a discrepancy to the learned mappings and measured perceptual biases. If participants learn to integrate hand movement distance and the quantity of random objects, then mutual perceptual biases should be observed. That is, an increase in the number of dots for a certain movement distance should enlarge/shorten the perceived movement distance for Group A/Group B in Experiment 1. Conversely, an increase in hand movement distance for a certain number of dots should increase/decrease the perceived number of dots for Group A/Group B in Experiment 2.

## Methods

### Participants

Right-handed participants were recruited through the participant pool (SONA systems) of the University of Würzburg. All of them had normal or corrected-to-normal vision and received monetary compensation for their participation. All participants provided written informed consent before participation. The study has been approved by the local ethics committee (Ethikkommission des Institutes für Psychologie der Humanwissenschaftlichen Fakultät der Julius-Maximilians-Universität Würzburg, GZ 2019-04) and the methods were performed in accordance with the relevant guidelines and regulations. Experiment 1 included 33 females and 15 males (age: *M* 24 years, *SD* 4) and Experiment 2 included 37 females and 11 males (age: *M* 26 years, *SD* 6). These sample sizes ensured a power of 0.95 (α = 0.05) for effect sizes of about dz = 0.5.

### Apparatus

The experiments were performed in a dimly lit experimental room. Participants were seated in front of a 19′ monitor (Fujitsu Siemens P19-1; 1280 × 1024 pixels; 1 pixel = 0.294 mm; 60 Hz) placed at about 105 cm distance. A chin rest supported their heads (see the left upper part of Fig. 1). The right hand was used to manipulate a movement device that was composed of a graphics tablet (Intuos 4 A4, Wacom), a digitizing stylus and a pincer like construction that held the stylus up on the tablet and enabled hand movements forward and backward along a track. Participants placed their fingers on two U-shaped plastic plates, which were part of the pincer construction. The index and the middle fingers were placed on one plate, the thumb on the other. A black cover prevented the vision of the hand and of the movement device. Participants pressed buttons of a computer mouse with the left hand to indicate their perceptual decisions. Auditory stimuli were initially presented through headphones. Later, due to local pandemic regulations, loudspeakers were used.

### Stimuli and trial procedure

The main trial events are shown in the lower part of Fig. 1. Before each trial, participants placed their hand at the location of a mechanical stop close to their body (i.e. at a start location). Then a visual prompt (“New trial” in German; in light grey) and a short beep tone indicted the beginning of a new trial. In response to this start signal, participants had to move a green cursor (a dot of 3 mm in size) that appeared at the lower part of the screen towards the center of a black square (4.8 × 4.8 cm) that was presented at the screen’s center. The cursor movement was controlled by the movement of the hand so that if the hand moved forward/backward the cursor moved up/down. There were no specific constraints regarding movement velocity except that the participants were asked to not move too fast (to not damage the apparatus) as well as too slow (to not lengthen the duration of the experiments). When the center of the square was reached (i.e. when the spatial deviation between the cursor position and the center of the square was smaller than 2.94 mm) the cursor disappeared and the square was filled with a number of black randomly distributed dots (1.2 mm in size). Simultaneously, a durable clicking noise was presented (additional feedback that the movement goal was reached) and the participants were required to maintain this body state for 1 s. If the hand location changed during this period (i.e. if the spatial deviation between the cursor position and the center of the square exceeded 2.94 mm) the dots disappeared, the cursor reappeared, and the participants had to perform corrective movements. Then, participants had to move the hand back to the start position. During this backward movement, the cursor was not visible. Reaching the start position was accompanied by a short beep tone that was also a signal to initiate the second movement to the center of the square. This second movement phase corresponded to the first movement phase (except for stimulus and movement features that are described below). Finally, a blue question mark appeared and the participants had to judge either whether the first or the second movement distance was larger (in Experiment 1) or whether the first or the second visual stimulus contained more dots (in Experiment 2). The left mouse button was assigned to the first stimulus/movement, the right mouse button to the second.

### Design

**Figure 2:**
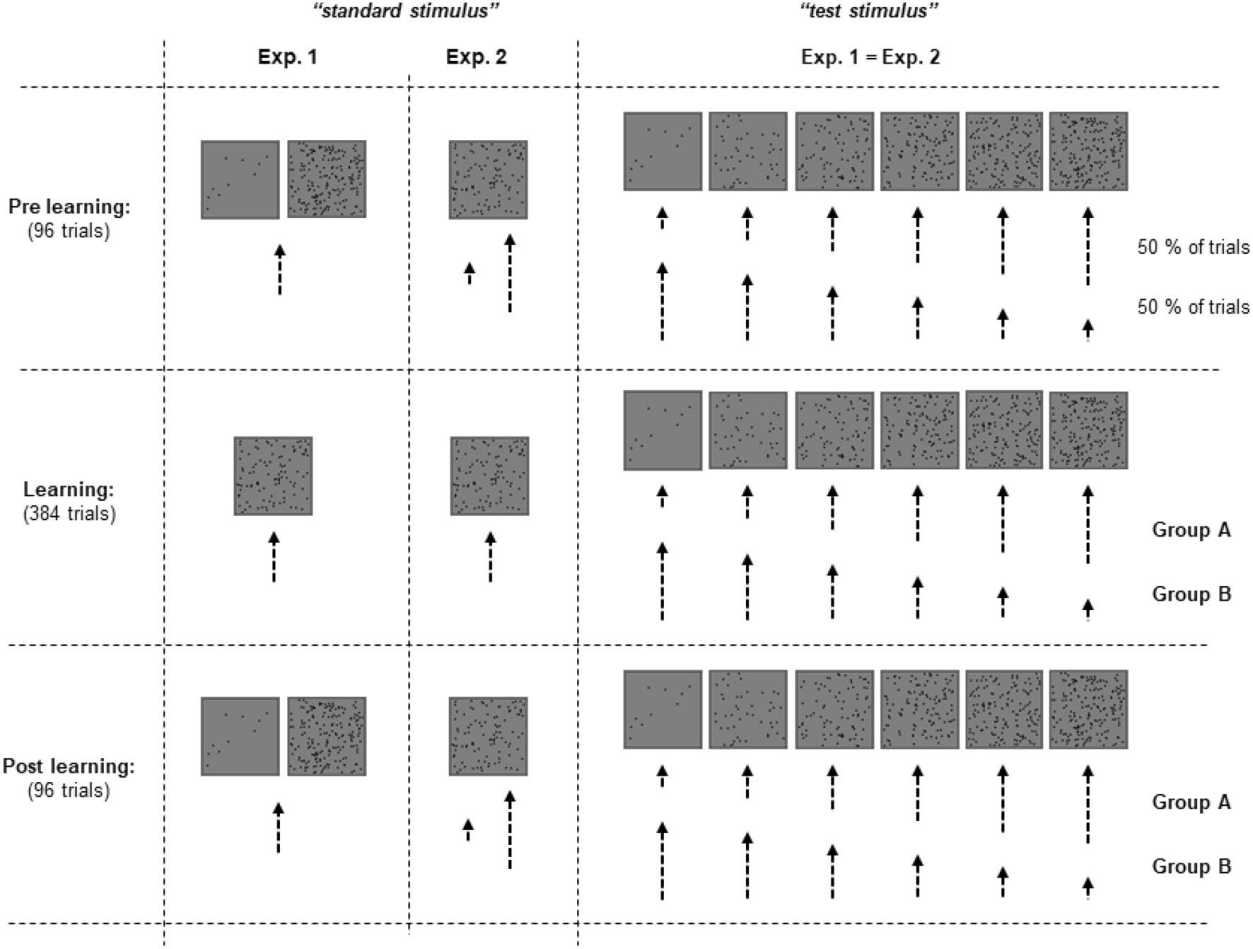
Assignment of hand movement distance to the number of dots for the standard and test stimuli in Exp.1 and Exp.2. The length of the dotted lines indicates the spatial extent of movement distance covered by the hand. The shortest line stands for about 7 cm, the largest for about 15 cm. The middle line on the left part of the figure indicates an intermediate distance (11 cm). The number of dots varied between 10 and 190, and was 100 for the standard stimulus of Exp.2 (as well as of Exp.1 in the learning phase). See main text for more details.

We used a method of constant stimuli so that one virtual interaction with the visual object served as a standard stimulus, another one as a test stimulus (random assignment to first and second movement phases). Each experiment was divided in three phases: a pre-learning (one block of 96 trials), a learning (4 blocks of 96 trials each) and a post-learning phase (one block of 96 trials), which were preceded by 16 practice trials (not included in the analyses). One important experimental manipulation in both experiments was related to the spatial extent of hand movement required to reach the square’s center. That is, we varied the transformation of the hand movement distance to the cursor movement distance (i.e. gain). Another important manipulation was related to the number of dots appearing during the first and the second movement phases. The differences between both experiments are described below (see also Fig. 2).

#### Experiment 1

Here we were interested in how the perception of the body rather than visual perception of an object is affected by visuo-motor learning. Thus, the spatial extent of hand movements served as a standard and a test stimulus. In particular, the spatial extent of one of the hand movements was always about 11 cm (standard stimulus) and corresponded to a 1:1 mapping between cursor and stylus movements. This “standard movement” was paired with the square that contained either a rather small (10) or a rather large number (190) of dots in the pre-learning and post-learning phases. In the learning phase, an in-between number of dots was used (100). The distance of hand movements that served as a test stimulus varied between 7 and 15 cm in six equidistant steps of 1.6 cm [These values are “ideal” values that we aimed by the gain manipulation. The actually measured distances amounted to 7.25 (SD 0.04), 8.85 (SD 0.04), 10.45 (SD 0.05), 12.03 (SD 0.05), 13.62 (SD 0.05) and 15.20 (SD 0.05) cm. These distances were thus slightly larger on average (M 0.23) than the ideal values. Moreover, this constant measurement error slightly decreased with an increase in movement distance from 0.25 (7 cm condition) to 0.20 (15 cm condition). These small deviations from the aimed values can be assumed to not substantially influence the results and drawn conclusions. The only implication is that the scale of the test values used for analyses is very slightly distorted (by maximally 0.5 mm)]. These “test movements” were assigned to the number of dots in the visual stimulus so that an increase in movement distance was associated either with an increase (“mapping A”) or with a decrease (“mapping B”) in the number of dots. The number of dots for these test movements varied between 10 and 190 in equidistant steps of 36 dots. Both mappings were used with equal probability for each participant in the pre-learning phase. In the learning and post-learning phases, each participant experienced either mapping A or mapping B depending on whether he/she was assigned to Group A or B (counterbalanced across participants). The main question of interest was how the judgment behavior differs between the different numbers of dots associated with the standard movement in the post learning phase depending on visuo-motor learning experience (i.e. on the group membership).

#### Experiment 2

Here, the experimental logic was partly reversed because we were now interested in how visual perception of an object rather than the perception of the body is affected by visuo-motor learning. One of the filled squares now served as a “standard stimulus”. It always contained 100 dots. Another filled square was the “test stimulus”. The number of dots in the test stimulus varied around the number of dots in the standard stimulus from 10 to 190 in equidistant steps of 36 dots. The gain was adjusted so that hand movements to the standard stimulus were either rather short (approx. 7 cm) or rather long (15 cm) in the pre-learning and post-learning phases. In the learning phase, an in-between distance was used (11 cm). The distance of hand movements aimed at the test stimulus again varied between 7 and 15 cm in six equidistant steps of 1.6 cm. These distances were assigned to the number of dots in the visual stimulus in the same way as in Exp. 1. That is, an increase in movement distance was associated either with an increase (“mapping A”) or with a decrease (“mapping B”) in the number of dots. In the pre-learning phase, both types of this assignment were used with equal probability for each participant. In the learning and post-learning phases, in contrast, each participant exclusively experienced either one or another type. That is, each participant was randomly assigned either to Group A that experienced mapping A or to Group B that experienced mapping B, as in Exp.1. The main question of interest was whether and how the judgment behavior differs between the short and long movements towards the standard stimulus in the post learning depending on visuo-motor learning experience (i.e. on the group membership).

Both experiments were thus identical except for the judgment type when only the test stimulus is considered. They differed basically in whether two different visual stimuli were assigned to a single movement (Exp.1) or two different hand movements were assigned to a single visual stimulus (Exp.2) when the standard stimulus is considered (see Fig. 2). This intended difference enabled us to examine the impact of visual stimuli on body perception (i.e. felt movement amplitude) and, vice versa, of body-related variables on visual perception holding the crucial physical stimulation unchanged across the levels of the independent measure.

### Data analyses

The main measure of interest was the point of subjective equality (PSE) computed as the level of the test stimulus at which the test stimulus was chosen with a frequency of 50%. We used a local model-free fitting procedure^28^ to fit proportions of trials in which the test stimulus was judged as “larger” (i.e. as having a larger movement amplitude in Exp. 1 and more dots in Exp.2) as a function of the test stimulus for different characteristics of the standard stimulus, and both learning groups in the pre- and post-learning phases. The data as well as program scripts (E-prime, Psychology Software Tools, Pittsburgh, PA) have been made publicly available (https://osf.io/hcgfr/).

Five participants of Exp. 1 and one participant of Exp. 2 were excluded from further analyses due to a very low discrimination performance in at least one of the critical conditions (the slope of the psychometric function was close to zero or even negative).

### Hypothesis

An increase in the number of dots for the standard movement in the post learning phase of Exp.1 was expected to increase the PSE for Group A, but to decrease the PSE for Group B. In a similar vein, an increase in hand movement distance for the standard stimulus in the post learning phase of Exp.2 was expected to increase the PSE for Group A, and to decrease the PSE for Group B. These interaction patterns would suggest sensory integration of visual and body-related signals following learning of arbitrary mappings between them.

## Results

**Figure 3:**
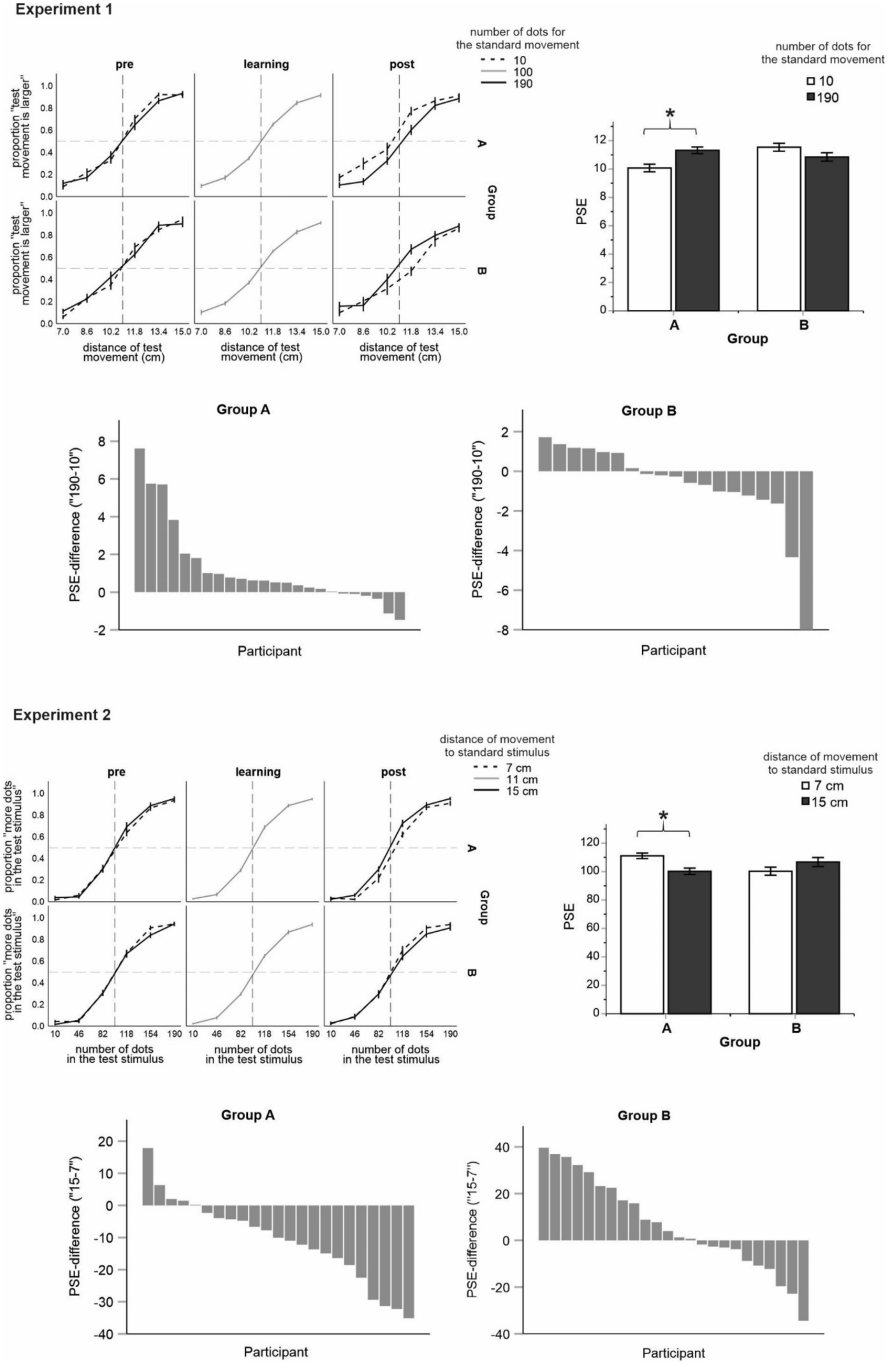
Results of Exp.1 (upper part) and Exp.2 (lower part). Shown are mean judgment data for all conditions (line graphs), mean PSE values for the post learning phase (graphs including white and black bars), and the effects (i.e. PSE differences) of the varying number of dots (Exp.1) and of the varying movement distance (Exp.2) on judgment behavior for each individual participant (graphs including gray bars sorted by their magnitude). Error bars are standard errors. Asterisks denote statistical significance (p < 0.05).

Mean judgment data and the corresponding PSE values are shown in Fig. 3. In Exp.1, an analysis of variance (ANOVA) of PSEs including number of dots as a within subject variable and group as a between subject variable revealed a significant interaction between both factors, *F*(1, 41) = 7.81, *p* = 0.008, *η*_*p*_^2^ = 0.160. Pairwise comparisons (t-tests) further showed that the effect of the number of dots was significant for Group A, *t*(23) = 2.72, *p* = 0.012, but not for Group B, *t*(18) = 1.32, *p* = 0.202 (see also Fig. 3 for the magnitude of the effects for each participant). The PSE significantly increased for Group A and only descriptively decreased for Group B when the number of dots associated with the standard movement increased in the post learning phase. Thus, the main hypothesis (of sensory integration) was supported by the results, however, only partially as the effect for Group B was not significant.

In Exp.2, an analysis of variance (ANOVA) of PSEs including movement distance as a within subject variable and group as a between subject variable revealed a significant interaction between both factors, *F*(1, 45) = 12.02, *p* = 0.001, *η*_*p*_^2^ = 0.211. Pairwise comparisons further showed that the effect of the number of dots was significant for Group A, *t*(22) = 3.94, *p* < 0.001, but not for Group B, *t*(23) = 1.57, *p* = 0.130. The PSE significantly decreased for Group A and only descriptively increased for Group B when movement distance to standard stimulus increased in the post learning phase. This pattern of results was of an opposite direction compared to what we predicted and what we observed in Exp. 1.

We also tested whether such PSE differences could be present before learning. For this purpose we performed the same analyses for the pre learning phase. The characteristics of the standard stimulus or movement did not interact with Group in these analyses, *F*(1, 41) = 0.17, *p* = 0.681, *η*_*p*_^2^ = 0.004 and *F*(1, 45) = 1.31, *p* = 0.259, *η*_*p*_^2^ = 0.028 for Exp.1 and Exp.2 respectively (all other *p* values > 0.376). These results support the view that the observed changes in judgment behavior observed in the post learning phase were in fact due to learning of visual and body-related task characteristics.

Finally, we tested whether the samples of participants of both experiments differed in regard to mean age and to the number of women and men, what, in theory, can lead to different outcomes of both experiments. This was not the case, *t*(45) = 1.19, *p* = 0.242 (for age differences) and *χ*^2^ (1) = 0.341, *p* = 0.559 (for sex differences). Thus, possible age or gender differences cannot account for the different results of Exp.1 and Exp.2.

## Discussion

The results of Experiment 1 revealed that a group of participants that learned to expect more dots with larger hand movements judged the movements made to targets with many dots as longer than the same movements made to targets with few dots. In another group of participants that was trained with an opposite association (more dots & smaller movements) no significant effects of the number of dots on the judgments of hand movement distance were observed. In Experiment 2, a group of participants that learned to expect more dots with larger hand movements judged the same number of dots as smaller when larger rather than smaller hand movements were executed. In another group of participants that was trained with an opposite association (more dots and smaller movements) no significant effects of the hand movement distance on the judgments of the number of dots were observed. We first discuss the significant effects observed in participants who learned to associate more dots with larger hand movements and then touch on why there were no significant effects in participants who learned an opposite mapping.

The main result of Experiment 1 was a change in the perception of body-related signals after a conflict was introduced relative to an arbitrary mapping of these and visual signals learned before (i.e. after learning that more dots are associated with larger movements). This outcome is in line with previous similar reports^19–22^ and indicates that participants learned to integrate signals that were unrelated before learning, and still integrated them after an intersensory conflict was introduced. In essence, it suggests that the body-related signals associated with hand movements and the visual stimulus information were treated as redundant (or as features of a common event) to some extent after learning and this led to a coupling of these signals in perception. Sensory integration is usually construed as a process of weighted signal averaging with weights being proportional to signal precision e.g. Ref.^12^. If there is a discrepancy between the signals their averaging (i.e. integration) inevitably produces perceptual biases. The main result observed in Exp.1 presumably represent such a bias. The results of Experiment 2, however, revealed a perceptual bias of the opposite direction following a very similar setup as the same number of dots was judged as smaller (rather than larger) when larger movements were executed. This outcome indicated that the signals were kept separate rather than being integrated.

The main difference between the experiments was the judgment task that stressed either body-related (Exp.1) or visual (Exp.2) signals. One factor possibly responsible for the different outcomes might be attention devoted to these signals. Participants certainly allocated more attention to the modality that was emphasized by the instruction. Importantly, attending the visual signals more than body-related signals presumably entailed a multimodal integration of these signals, whereas attending the body-related signals more than visual signals resulted in a perceptual segregation of the these signals. To understand how such an outcome could emerge consider that focusing attention on one modality often decreases multimodal integration compared to conditions in which attention is more evenly distributed across modalities^29–31^. This likely occurs because the expectation that multimodal signals belong together (i.e. the “unity assumption”) is weakened if only one modality is attended^32^. Thus, the way attention is distributed seems to be closely related to the participants’ assumption about the origin of the multimodal signals that determines their decision to integrate or not to integrate. Decisions to integrate seem more favored if attention is more broadly distributed across the signals. Based on this, our results indicate that under the present conditions participants believed to a lesser extent that the visual and body-related signals belong together when the judgment task directed their attention to vision rather than to their body. This could be so because body-related signals could better be ignored when the judgment task was visual in nature as compared with the role of vision in the judgments of hand movements^31^. As a result, attention was shared between the modalities more in the body-related than in the visual task. Such an attentional asymmetry could arise from a higher saliency of visual than body-related information in the visuomotor task we used (see also Ref.^4^).

There are, of course, also other possible explanations for the apparently opposite patterns of results observed in Exp.1 and 2. For example, irrespective of a possible role of attentional distribution, causal inference processes (i.e. unity assumption) could, in theory, be directly related to the precision of a currently attended signal. That is, attending a less precise (e.g. proprioceptive) signal could generally increase the readiness to integrate than attending a more precise (e.g. visual) signal. In addition, although the samples of participants of both experiments were comparable with respect to the mean age and the number of males and females (see “Results”) there could still have been differences between the samples with respect to other characteristics. For example, participants of Exp.2 could be less sensitive to their body-related signals than the participants of Exp.1. As a result, the body-related signals were rather ignored in Exp.2, and did thus not enter multisensory integration. More research is needed to better evaluate these and related possibilities.

Regarding the type of the implemented mappings we observed significant perceptual biases when an increase in hand movement distance was associated with an increase in the quantity of visual objects. In contrast, when an increase in hand movement distance was paired with a decrease in the quantity of objects, only descriptive trends in the expected directions were evident. This outcome likely relates to the disposition or preparedness to associate certain stimuli more than others^33^. A large stimulus magnitude in one modality seems to be more easily paired with a large rather than with a small stimulus magnitude in another modality. This apparent learning disposition is also an indicator of the involvement of a common representational system for magnitude in the brain during multimodal learning^34^.

Given the obvious artificiality of the experimental situation we examined one could wonder how the present results and conclusions can be applied to daily life. First of all, basic research on fundamental processes of perception and action almost always comes with paradigms that barely resemble specific real life scenarios to facilitate experimental control. Yet, our result indicate that our perception of own body and of external objects can change under certain condition even though the body and objects do not change, and one might think of real life scenarios where this happens. For example, when playing a string instrument musicians acquire a certain relationship between tactile feedback and sound intensity. If now that relationship changes (e.g. when playing in a room with more dampened acoustics) the present finding would suggest that both, auditory and tactile perception change, such that e.g. a certain objectively constant force on the instrument is felt as weaker in a room that goes with reduced auditory feedback from the instrument. Also, consider, e.g., a basketball player who practices throwing a ball into a basket again and again, or a mountaineer who repeatedly ascents mountains wearing a backpack of a varying weight. The present results and our rationale would suggest that after a certain amount of practice certain body-related characteristics of the basketball player and the mountaineer, and certain correlated characteristics of the objects (such as of a basket and of a hill) can be associated. As a result, changes in object and body perception could emerge under certain conditions (i.e. if the situation causes a kind of a multisensory discrepancy). Such observations are in fact reported^23,24^ and we treated them in more detail elsewhere^27^ (see also “Introduction”).

The present research can also be considered in the context of studies on perceptual-motor adaptation (or (re-)calibration) that are discussed with reference to the Gibsonian ecological approach e.g. Ref.^35^, for reviews see Refs.^36,37^. According to this line of research the perceptual and motor systems are attuned or calibrated to each other during learning to successfully act in a particular situation. Changes in either the perceptual or motor system, e.g. due to growth, changing environmental conditions or due to using of different tools, can then lead to disturbances of such a mapping and consequently, to inaccurate actions. In response to such a disturbance the originally learned mapping is updated (or re-calibrated). Following this reasoning in terms of different stages of a dynamic process of perceptual motor adjustments e.g. Refs.^37,38^, the learning part of the present experiments can be considered as an attunement or calibration stage, whereas the post-learning part included a disturbance of the learned mapping. As we measured the perception of movements and visual stimuli in response to the disturbance, our results can be indicative of perceptual changes accompanying re-calibration.

To conclude, the present study suggests that originally unrelated characteristics of observers’ body movements and of environmental objects can be learned following a co-occurrence of that characteristics. This learning can give rise to changes in the perception of own body and of external objects when a discrepancy to the learned relation is introduced.

## Data Availability

The data as well as program scripts have been made publicly available via the Open Science Framework and can be accessed at https://osf.io/hcgfr/.
